# Consequences of radiopharmaceutical extravasation and therapeutic interventions: a systematic review

**DOI:** 10.1007/s00259-017-3675-7

**Published:** 2017-03-16

**Authors:** Jochem van der Pol, Stefan Vöö, Jan Bucerius, Felix M. Mottaghy

**Affiliations:** 1 Department of Radiology and Nuclear Medicine, Maastricht University Medical Centre (MUMC+), Postbox 5800, 6202 AZ Maastricht, The Netherlands; 2 Department of Nuclear Medicine, University Hospital, RWTH Aachen University, Pauwelsstr. 31, 52072 Aachen, Germany

**Keywords:** Extravasation, Dose infiltration, Radiopharmaceuticals, Radiation ulcer

## Abstract

**Purpose:**

Radiopharmaceutical extravasation can potentially lead to severe soft tissue damage, but little is known about incidence, medical consequences, possible interventions, and effectiveness of these. The aims of this study are to estimate the incidence of extravasation of diagnostic and therapeutic radiopharmaceuticals, to evaluate medical consequences, and to evaluate medical treatment applied subsequently to those incidents.

**Methods:**

A sensitive and elaborate literature search was performed in Embase and PubMed using the keywords “misadministration”, “extravasation”, “paravascular infiltration”, combined with “tracer”, “radionuclide”, “radiopharmaceutical”, and a list of keywords referring to clinically used tracers (i.e. “Technetium-99m”, “Yttrium-90”). Reported data on radiopharmaceutical extravasation and applied interventions was extracted and summarised.

**Results:**

Thirty-seven publications reported 3016 cases of diagnostic radiopharmaceutical extravasation, of which three cases reported symptoms after extravasation. Eight publications reported 10 cases of therapeutic tracer extravasation. The most severe symptom was ulceration. Thirty-four different intervention and prevention strategies were performed or proposed in literature.

**Conclusions:**

Extravasation of diagnostic radiopharmaceuticals is common. ^99m^Tc, ^123^I, ^18^F, and ^68^Ga labelled tracers do not require specific intervention. Extravasation of therapeutic radiopharmaceuticals can give severe soft tissue lesions. Although not evidence based, surgical intervention should be considered. Furthermore, dispersive intervention, dosimetry and follow up is advised. Pharmaceutical intervention has no place yet in the immediate care of radiopharmaceutical extravasation.

## Introduction

High doses of radiation exposure can potentially cause severe tissue damage, such as skin desquamation and necrosis. Extravasation of radionuclides used in nuclear medicine practice results in localized tissue retention of the radiopharmaceutical and subsequently in an unintended extended local radiation exposure. Because of the character of the radiation, extravasation of therapeutic radiopharmaceuticals has the highest tendency to result in tissue damage, although some cases of tissue damage following the extravasation of diagnostic radiopharmaceuticals have been reported [1].

Knowledge of possible consequences and interventions to prevent tissue damage are vital for an adequate risk-adapted management after extravasation of radiopharmaceuticals. The EANM procedure guideline for 90Y-radiolabeled ibritumomab tiuxetan (Zevalin®) is the only guideline that gives limited practical advice in case of extravasation, advising local hyperthermia, elevation of the extremity and gentle massage [2]. The SNMMI procedure standard for palliative treatment for painful bone metastases advises local heat to promote reabsorption [3]. Other EANM and SNMMI guidelines covering radionuclide therapy do not give any practical information in case of extravasation, regardless of the potential complications [4–7].

To our knowledge, no previous study or literature review has been performed to summarize the effects of the extravasation of commonly applied diagnostic or therapeutic radiopharmaceuticals. Knowledge of the incidence of extravasation, the severity of these effects, and about the effectivity of interventions is necessary for adequate clinical response in case of extravasation, as well as in development of guidelines covering radiopharmaceutical extravasation. The purpose of this study was, therefore, to review systematically previously published data on the incidence and clinical outcome of radioactive extravasations and to summarize the reported incidences of events of most of the clinically used radiopharmaceuticals, the applied interventions, as well as the reported clinical outcomes.

## Materials and methods

### Search strategy

A computer-aided search of the PubMed/MEDLINE and Embase databases was conducted to find relevant published articles on extravasation of radiopharmaceuticals. No start date limit was used. No language limitation was applied in the initial search strategy. The search string was composed of several synonymous keywords for extravasation combined as a group using the “AND” operator with a combination of the keywords “radiopharmaceutical” and an extensive list of clinically used isotopes in five different notations (i.e. “I-131”, “I131”, “131I”, “Iodine 131”, “131 iodine”). In PubMed, all keywords were combined with a MESH equivalent when available, as well as an equivalent with the “Pharmaceutical action” tag. The used search strings are shown in Table 1. The search was updated until November 2016.

**Table 1:** Search strings applied in PubMed/MEDLINE and Embase with search results specified to search engine and search strings

No.	Search strings^a^	Pubmed/MEDLINE	Embase
1	“Extravasation of Diagnostic and Therapeutic Materials” [Mesh] OR “Extravasation of Diagnostic and Therapeutic Materials” OR “extravasation” OR “infiltration” OR “misadministration”	110.807	171.853
2	“I-123” OR “I-124” OR “I-125” OR “I-131” OR “Tc-99m” OR “F18” OR “Ga-68” OR “In-111” OR “Tl-201” OR “Rb-82” OR “N-13” OR “O-15” OR “C-11” OR “Er-169” OR “Re-186” OR “Sr89” OR “Sm-153” OR “Y-90” OR “Ra-223” OR “P-32” OR Lu177	213.922	361.049
3	“Radiopharmaceuticals”[Mesh] OR “Radiopharmaceuticals” OR “Radioisotopes”[Mesh] OR “Radioisotopes”	301.588	15.949
4	1 AND (2 OR 3)	2.153	3.493

Search strategy with number of yielded results in PubMed/MEDLINE and Embase.

The “Mesh” tag was omitted for the Embase search strings

^a^In the actual search strings used, all radionuclides were spelled using five different conventions, i.e. the keyword “I-123” was accompanied by “I123”, “123I”, “Iodine 123”, and “123 Iodine”, grouped together with the “OR” operand

Studies reporting radiopharmaceutical extravasation in humans were eligible for inclusion. Only studies written in the languages mastered by the authors were included: English, Spanish, French, Italian, German, Hungarian, Romanian, or Dutch. No other limits were imposed. Animal studies were excluded.

Two reviewers (J.P. and S.V.) independently reviewed titles and abstracts to find articles reporting cases of extravasation, or to find literature otherwise relevant to the subject. In case of disagreement on relevance, the full text was retrieved. To expand our search, bibliographies of articles that finally remained after the selection process were screened for potentially relevant references. Subsequently, the corresponding full text articles were retrieved for further reading and selection.

### Data extraction

The following data were extracted whenever available from eligible studies: first author, journal and year of publication, population studied, number of reported extravasations, radiopharmaceutical, injection place, estimated administered volume, estimated extravasated activity and tissue dose, description of tissue damage and delay since injection, duration of follow-up, and applied medical interventions.

### Data analysis

Reported incidents were categorized and pooled according to radiopharmaceutical that was administered. Ratios were calculated between the pooled reported incidence of extravasation versus the number of reported incidences of adverse soft tissue effects. Cases were grouped and displayed in tables sorted by radiopharmaceutical separately for diagnostic and therapeutic radiopharmaceuticals. Furthermore, literature references that described interventions were organised in three categories: 1) advised by reference, 2) applied in case report, and 3) discouraged by reference.

## Results

The searches performed using Pubmed and Embase resulted in 2153 and 3493 publications respectively (Table 1). Of these, 1123 search results were found using both search engines yielding a total of 4523 abstracts after subtraction. Rejected abstracts described irrelevant animal studies (1012), reported extravasation of other agents than radionuclides (198), mentioned infiltration of other nature than infiltration of radiopharmaceuticals (2424), described lymph drainage studies or other nuclear medicine studies in which extravasation is a pathological finding, i.e. urinary extravasation in ^99m^Tc-mercaptoacetyltriglycine (MAG3) (196), or publications about radionuclide use for other purposes than nuclear medicine studies (603), such as radioimmunoassays. The full text was retrieved for the remaining 81 publications. The references of relevant publications were screened, of which the full text was retrieved. In total, 108 full text articles or conference abstracts were retrieved for further evaluation. Radiopharmaceutical extravasation was reported in 44 publications, of which 37 about diagnostic and eight about therapeutic radiopharmaceuticals. Another 10 publications contained information on extravasation based on expert opinion or cited work.

Cases of diagnostic extravasation are summarised in Table 2. In total, 37 publications reported 3016 cases of radiopharmaceutical extravasation. For three cases symptoms and follow up was reported (0,1%) [42–44]. When grouped together, a total of 3003 cases described extravasation without reported symptoms after extravasation of 18F-fluorodeoxyglucose (FDG) (332) and 99mTc-labelled tracers (2671) [8–41]. Radiation ulcers in two patients following extravasation of 201Tl-thallous chloride were the most severe injuries reported [43, 44]. In one case a radiation ulcus was diagnosed 2 years. The injected activity and estimated tissue dose were 74 MBq and 200 Gy, respectively [43]. In the second case the diagnosis radiation ulcus was made after 3 years. The injected activity was 111 MBq and the worst case estimate of tissue dose was 250 Gy [44]. A pruritic and erythematous patch was described following the extravasation of 34 MBq of 131I-iodocholesterol, with a worst case tissue dose estimate of 490 Gy [42]. Other reported cases of diagnostic extravasation cases did not describe dosimetric parameters or follow-up.

**Table 2:** Summary of reported cases of diagnostic radiopharmaceutical extravasation

References	Total reported cases	Radiopharmaceutical	No. of patients with reported radiation injury	No. of patients with reported follow-up	Most severe injury reported
[8–17]	332	18F-FDG	0	0	
[18–31]	2584	^99m^Tc bone tracers	0	0	
[32]	3	^99m^Tc-MAA	0	0	
[33]	1	^99m^Tc-DMSA	0	0	
[34, 35]	10	^99m^Tc-DTPA	0	0	
[36]	1	^99m^Tc-HMPAO	0	0	
[37]	1	^99m^Tc-MAG3	0	0	
[19, 38, 39]	15	^99m^Tc-pertechnetate	0	0	
[40, 41]	2	^99m^Tc-sestamibi	0	0	
[19]	38	^99m^Tc-sulfurcolloid	0	0	
[19]	16	^99m^Tc-microspheres	0	0	
[42]	1	^131^I-iodocholesterol	1	1	Erythematous plaque and pruritus.
[43–45]	12	^201^Tl-thallous chloride	2	2	Radiation ulcer
Total	3016		3	3	

Eight publications reported a total of 10 cases of therapeutic radiopharmaceutical extravasation [46–53]. Radionecrosis was the most severe symptom reported in five cases [47, 49, 50], although three cases reported needle track necrosis that resolved spontaneously [47]. The results and references are summarised in Table 3. Table 4 summarises a total of 34 interventions that are advised or, contrarily, discouraged in literature and those which are applied in reported cases.

**Table 3:** Summary of reported cases of therapeutic radiopharmaceutical extravasation

Reference	No. of patients with extravasation	Radiopharmaceutical	Reported extravasated activity [GBq]	Reported administered volume [ml]	Reported estimated tissue dose [Gy]	Symptoms after radiopharmaceutical extravasation^a^
Williams 2006 [52]	1	^90^Y-ibritumomab tiuxetan	0,068–0,136	60	10–20 worst case	Erythema (1d), tenderness (14d), bulla (26d), moist desquamation (29d)
Siebeneck 2008 [50]	1	^90^Y-ibritumomab tiuxetan	Not reported	Not reported	Not reported	Small erythematous area (1w). Progression to 15 × 25 cm erythematous area (4w). Moist desquamation (5w). No healing progression after 8-15w. Skin graft was advised. After 4m start of tissue granulation, with greyish necrotic in the centre size of dime.
Erken 1991 [47]	3	^90^Y-colloid (radiosynovectomy)	Not reported	Not reported	Not reported	Needle track necrosis. Spontaneous healing (3m)
Terwinghe 2012 [51]	1	^90^Y-DOTATOC	3,5 (worst case)	Not reported	Not reported	Painful and swollen arm (p.i.). No symptoms arose during follow-up (no time indication).
Minsky 1987 [48]	1	^32^P-sodium phosphate	0,086	76	5,02	Raised area at infusion site (p.i.).
Patton 1950 [49]	1	^90^Y-hydroxy citrate complex	Not reported	0,2	1000	Ulceration, 2cm in diameter.
Bonta 2011 [46]	1	^131^I-metaiodobenzylguanidine	11,1 (worst case)	60	20–40	Forearm swelling (7d). Rash at injection site, 10x5cm (4w). Lesion still “angry looking” (7w), lesion appearance evolved to dry and scaly after corticosteroid cream.
Kawabe 2013 [53]	1	^89^Sr-Strontium chloride	0.00296	30	1.78	Slight burning pain, slight reddening and small circular swelling. No symptoms reported during follow up.

^a^Whenever available, the time of symptom presentation and other events is printed between brackets, the following abbreviations are used: *d* days, *w* weeks, *m* months, *p.i*. post injection

**Table 4:** Summary of interventions advised in literature or applied in reported cases

Category	Intervention	Advised by references			Reported to have been applied	Discouraged by reference
		Diagnostic	Therapeutic	N.S.^a^		
General	Immediate cessation of the administration	[1, 34]	[1, 2, 34, 46, 51, 52, 54]	[55]	[52]	
	Aspiration of venous IV-catheter	[1, 34]	[1, 34]			
Concentrating	Cooling extravasated region			[1]		
Dispersive	Warming extravasated region	[1, 34, 56]	[1–3, 34, 46, 52]	[55, 57, 58]	[48, 53]	[59]
	Arm elevation	[34]	[2, 34, 46, 54]		[51]	
	Massage	[1]	[1, 2, 54]	[55, 58]	[51, 53]	
	Compression stockings	[46]	[46]			
	Pressure application				[48]	
	Squeezing a stress ball				[51]	
Surgical	Saline flushing	[1]	[1, 34, 52]			
	Local puncture				[51]	
	Early excision and skin grafting		[49, 52]		[49]	
Pharmacotherapeutic	Intralesional steroids		[52]			
	Topical steroid application	[1]	[1]	[58]	[46, 53]	
	Diphenhydramine iv				[46]	
	Local penicillin application				[49]	
	Hyaluronidase	[1]	[1]	[55]	[45]	[52]
	Amifostine		[46]			
	Silver sulfadiazine				[50, 52]	
Organizational	Contain syringe	[34]	[34]			
	Clearance evaluation and dosimetry	[1, 34, 43, 57, 60]	[1, 34, 43, 57, 60]		[12, 42–44, 46, 48–53, 61]	
	Follow up	[43]	[1, 59]		[46, 47, 49–53]	
	Delineation	[1, 34]	[1, 34]			
	Report event	[1, 34]	[1, 2, 34]			
Preventive	Use of intravenous catheter,					
Port–a-cath or midline venous catheter	[56, 57]	[1, 2, 4, 46, 50, 51, 55–57]	[34]	[46, 51, 52]		
Measures	Dilution of radiopharmaceutical		[46]		[51, 53]	
	Administration under gamma camera		[59, 62, 63]		[59]	
	Catheter placement by Experienced technician		[1, 49, 52]			
	Choose a large vein between wrist and antecubital fossa for intravenous access		[51, 52]			
	Place intravenous access proximal to any venipuncture site established within 24h		[50–52]			
	Avoid antecubital fossa to minimize damage to vital structures		[1]			
	Check of patency	[1, 34]	[1–3, 34, 46, 52, 55]		[50, 53]	
	Dose rate ratio between injection site and corresponding contralateral site		[51]	[55]	[51]	
	Slow needle retraction while infusing anti-inflammatory agent^b^		[64]			

^a^Not specified

^b^Reported as preventive measure for needle track necrosis following radiosynovectomy

## Discussion

Multiple retrospective case series on bone scintigraphy, as well as 18F-FDG positron emission tomography (PET) report a large proportion of at least partial tracer extravasation [18–31]. Although there was no clinical follow-up after extravasation reported in these publications, no adverse reactions have been reported following extravasation of widely and frequently used ^99m^Tc labelled radiopharmaceuticals. Similarly, no cases have been found with any symptoms after extravasation of ^99m^Tc, ^123^I, ^18^F, and ^68^Ga labelled radiopharmaceuticals. These radiopharmaceuticals together comprise a great majority of radiopharmaceuticals in use on a daily basis in general nuclear medicine practices. Lack of clinical follow-up after diagnostic nuclear medicine scans, but also a conservative attitude towards reporting and publishing of complications may have possibly lead to under-reporting of skin lesions. Nevertheless, given the long history of frequent usage of these agents and even in case of significant under-reporting, we would have expected that at least a few cases had been reported. Therefore, we consider it safe to be conservative in treatment of extravasation of these tracers. Attention should only be focused on early complications of the extravasation that are not attributable to the radioactivity, such as skin necrosis and compartment syndrome [65, 66]. Other diagnostic radiopharmaceuticals were reported to cause at least mild skin lesions, notably ^201^Th-thallous chloride and ^131^I-iodine-iodocholesterol. Only a few publications report these cases, which are further elucidated by only limited provided data on dosimetric data, follow-up of the patients, etc. [42–44]. Particularly the long period of 2 and 3 years after ^201^Th-thallous chloride raise questions whether the skin lesions were radiation induced. Furthermore, it should be mentioned that ^131^I-iodine-iodocholesterol is used only sporadically nowadays. Nevertheless, in our opinion this warrants at least minimal preventive measures and follow-up after extravasation of these tracers.

Few complications following therapeutic extravasation were reported, yet some causing severe soft tissue damage. Considering the high prevalence of extravasation in diagnostic procedures, the same could be true for therapeutic radiopharmaceuticals. Nevertheless, it is plausible that generally more care is taken in preventing extravasation.

### Therapeutic options

Applied and advised interventions are mostly derived from treatment regimens of extravasation of non-radioactive agents. Only few were applied in reported cases. Dispersive actions can be effective in extravasation non-radiopharmaceutic agents [65, 66]. It can be debated if all listed dispersive interventions can be applied to radiopharmaceuticals. For instance, DeNardo argues that hyperthermia can ameliorate success of radiotherapy, similarly it might do more harm in case of extravasation [59]. On the other hand, it is plausible that warming up the tissue to promote hyperaemia and lymphatic flow might reduce the time of exposition enough to at least compensate this radiosensitising effect. Terwinghe et al. showed fast tissue wash out of 90Y-DOTATOC after arm elevation, warming the infiltrated area and squeezing a stress ball. This patient had no soft tissue symptoms during follow-up. Moreover they argue that the relatively low molecular weight contributes to faster tissue wash out, in comparison to radiopharmaceuticals with higher molecular weight, particularly in case of 90Y-ibritumomab tiuxetan [51]. Concentrating the radiopharmaceutical by cooling the tissue can be applied in anticipation of surgical interventions. Only two cases report the use of surgical techniques. Local puncture was not considered successful after extravasation of ^90^Y-dotatate [51]. In one report from 1950 the ulceration was excised [49]. Other surgical treatments have not been described or advised in literature. Pharmacotherapeutical interventions have been reported in sporadic case reports. Ulcers were treated with antibiotics and discomfort was treated with topical steroids [46, 49, 50, 52, 53]. Intralesional corticosteroid therapy is advised by Williams, based on results after chemotherapy extravasation, but has not been reported in radiopharmaceutical extravasation [52]. Hyaluronidase use is based on results in extravasation of other agents [1, 55], and applied in one case report of 201Tl-thallous chloride extravasation [45]. Williams et al. discourage any use, because of the experimental status [52]. Amifostine might be effective in radiopharmaceutical extravasation for its proven radioprotective properties in radiotherapy [46]. Despite, it remains unknown how it performs in the high linear energy transfer radiation environment of radiopharmaceutical extravasation, while having considerable side effects.

Clearance evaluation and dosimetry are often advised to be part of extravasation management. Different methods have been used, yielding a large range of tissue doses, due to uncertainties such as retained activity and the volume of the infiltrated tissue, as well as the use of worse case scenarios [44, 46, 48, 49, 52, 53, 60, 61]. Sequential activity measurements with probes or gamma-camera can give useful insight in biological half-life, as well as effectiveness of applied interventions [42, 51, 52]. Furthermore, it might be helpful to also estimate the amount, meaning the volume, of extravasation as one can assume, that larger volumes of radioactive extravasation might cause more pronounced side effects than smaller amounts. However, the volume of an extravasation is hardly measurable, at least in the clinical setting and, consequently, to define. This is not only because of the “real time” setting, but it is even more difficult based on a retrospective literature search and analyses. Furthermore, preventive measures are reported, such as the use of an intravenous catheter (IV-catheter) and adequate check of patency for both diagnostic as well as therapeutic extravasation [1, 2, 4, 46, 50, 51, 55–57].

### Limitations

A substantial number of publications reporting on extravasation, or which were otherwise relevant, were found by screening bibliographies and not in the initial search, despite the sensitive elaborate search strategy. This can be at least partially explained by mismatches in searched keywords and the subject of the publication. For instance, a number of publications about pitfalls in image interpretation contained a brief case report of extravasation as an example for false positive lymph node visualisation. These publications were filtered out in our initial search because they did not contain the right keywords in title, abstract or keyword index. Similarly, some patient studies investigating a particular tracer for evaluation of a specific pathology also report extravasation, but were also filtered out because no keywords relevant for our search were matched. Others were brief case reports or “image of the month”-type of publications without an abstract, but contained some relevant information in image captions. Finally, we found several congress abstracts for oral or poster presentations that were not indexed in PubMed or Embase. This is a minor shortcoming in our literature search, although the publications that were found this way only reported minimal information on tracer extravasation. Moreover, it is challenging to avoid such difficulties.

We did not analyse the effect of extravasation on the image quality of diagnostic nuclear medicine scans. It is obvious that image quality might significantly be hampered by at least large extravasation leading to a lower degree of tracer uptake in the target tissue (organ) and to the potential need for a new scan. However, as we, in this review, are focusing on clinical consequences for the patients, we do not address this issue in detail. Furthermore, because of the design of the study, gaining more insights into this topic is not possible within the context of this review.

### Future perspectives

The lack of data on interventions underlines the need for further scientific exploration on this subject. Future research is required to establish definite conclusions for all used radiopharmaceuticals, by retrospective or preferably prospective studies of extravasation cases with detailed clinical description, activity measurements, as well as serial scans to assess dynamic behaviour of the tracer in the time after extravasation. Furthermore, similar studies can be performed to evaluate the different therapies possible after extravasation. Alternatively, detailed case reports can proof to be helpful, especially for less used and probably less common extravasation of therapeutic radiopharmaceuticals. Nevertheless, trials performed in centres that treat large numbers of patients with nuclear medicine therapies are preferable.

### Local protocol

In our clinic, every injection of diagnostic and therapeutic radiopharmaceutical is performed via an IV-catheter, preceded by a check of patency that includes flushing the IV-catheter with saline solution, while visually inspecting if swelling occurs and asking the patient if he experiences discomfort during injection. Furthermore, some blood is drawn. When patency is doubtful, a second IV-catheter is inserted and checked for patency. Alternatively, in patients with difficult venous access, this step can be preceded by injecting ^99m^Tc-pertechnetate with the patient’s thorax positioned under a gamma camera, to visually confirm systemic spread. For therapeutic administrations, patency is always checked under supervision of the nuclear physician.

**Fig. 1:**
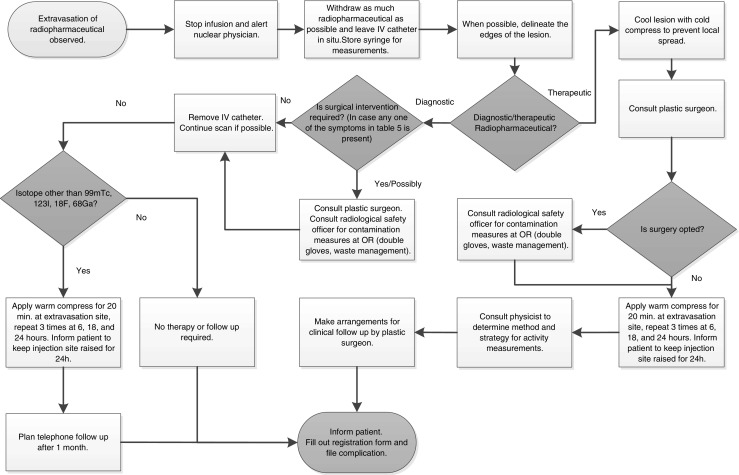
Flowchart describing the protocol in use in Maastricht University Medical Center for management of radiopharmaceutical extravasation

Figure 1 shows the protocol that is in use in our hospital for management of radiopharmaceutical extravasation. It is based on the findings of this review. It reflects the negligible probability of adverse events in frequently used ^99m^Tc, ^123^I, ^18^F and ^68^Ga labelled tracers by a conservative approach. A more careful approach has been chosen with relatively harmless preventive measures for diagnostic tracers combined with follow-up, in case of tracers for which adverse events have been reported, notably ^201^Tl-thallous chloride, and for tracers no literature of extravasation was found at all. Although in general radiopharmaceutical administration volume is limited, severe consequences have been reported in non-radiopharmaceutical extravasation, such as tissue necrosis and compartment syndrome [65, 66]. Therefore, in case of any of the symptoms listed in Table 5, the plastic surgeon is consulted. For therapeutic extravasation a plastic surgeon is always consulted to discuss the usefulness of surgery. Until the decision for surgical intervention is made, the lesion is cooled to spare surrounding tissue by preventing spread of the radiopharmaceutical. If no surgical intervention is opted, frequently warming the extravasation area and elevation of the arm are advised to promote spreading of the radiopharmaceutical. Repetitive gamma camera measurements are performed in case of therapeutic extravasation in consultation with the physicist. The patient should always be informed about treatment and potential complications. All cases of extravasation in our hospital are being recorded using a standard form containing detailed information, such as symptoms, the location of extravasation, injected volume and activity, as well as treatment. Furthermore, the incident is documented for the local complication committee. The strategy applied in this protocol ensures an efficient workflow, by minimizing the effort needed for the most frequently used tracers.

**Table 5:** List of symptoms requiring consultation of a plastic surgeon

Symptom
Swelling > 2.5cm in longest axis
Numbness
Blanched, translucent skin
Tight skin, leaking
Discolored, bruised skin
Circulatory or nervous impairment
Moderate-severe pain

Adapted from Amjad et al. [65]

## Conclusions

Extravasation of diagnostic radiopharmaceuticals is common. Often used ^99m^Tc, ^123^I, ^18^F, and ^68^Ga labelled tracers do not require specific intervention. Sporadic reports of extravasation of other diagnostic radiopharmaceuticals, however, have described soft tissue lesions. Dispersive intervention and follow-up is, therefore, advised in other diagnostic radiopharmaceuticals. Extravasation of therapeutic radiopharmaceuticals can lead to severe soft tissue lesions. Although not evidence based, surgical intervention should be considered. Furthermore, dispersive intervention, dosimetry and follow-up is advised. Pharmaceutical intervention has no place yet in the immediate care of radiopharmaceutical extravasation.
